# Reduced Corticosteroid Exposure Is Safe and Does Not Reduce Disease Control among Hodgkin Lymphoma Patients Treated with Escalated BEACOPP (eBEACOPP)

**DOI:** 10.3390/medicina60030430

**Published:** 2024-03-02

**Authors:** Ida Hude Dragičević, Sandra Bašić-Kinda, Helena Markotić, Martina Morić-Perić, Dino Dujmović, Ivo Radman, Barbara Dreta, Snježana Dotlić, Ivana Ilić, Lea Galunić Bilić, Margareta Dobrenić, Marko Kralik, Igor Aurer

**Affiliations:** 1 Department of Internal Medicine, Division of Hematology, University Hospital Center Zagreb, Kišpatićeva 12, 10000 Zagreb, Croatia; 2 Department of Internal Medicine, University Hospital Mostar, Ulica Kralja Tvrtka bb, 88000 Mostar, Bosnia and Herzegovina; 3 Department of Internal Medicine, General Hospital Zadar, Bože Peričića 5, 23000 Zadar, Croatia; 4 Department of Pathology and Cytology, University Hospital Center Zagreb, Kišpatićeva 12, 10000 Zagreb, Croatia; 5 School of Medicine, University of Zagreb, Šalata 2b, 10000 Zagreb, Croatia; 6 Department of Oncology and Radiotherapy, University Hospital Center Zagreb, Kišpatićeva 12, 10000 Zagreb, Croatia; 7 Department of Nuclear Medicine and Radiation Protection, University Hospital Center Zagreb, Kišpatićeva 12, 10000 Zagreb, Croatia; 8 Department of Radiology, University Hospital Center Zagreb, Kišpatićeva 12, 10000 Zagreb, Croatia

**Keywords:** Hodgkin lymphoma, eBEACOPP, corticosteroids, toxicity, avascular hip necrosis

## Abstract

*Background and Objectives*: eBEACOPP is the most effective chemotherapy regimen for younger patients with early unfavorable (EU) and advanced-stage (AS) Hodgkin lymphoma (HL), albeit with significant toxicities. The 14-day/cycle prednisone course contributes to side effects, including osteoarticular events like avascular bone necrosis (AVN). Our center has been using eBEACOPP since 2009 for AS and 2014 for EU patients. In 2016, we reduced prednisone treatment to 7–10 days to lessen AVN risk. We analyzed the effects of this approach. *Materials and Methods*: We retrospectively collected data on patients who received at least two cycles of eBEACOPP for first-line HL treatment. *Results*: A total of 162 patients (33 EU, 129 AS) were included. Their median age was 31 (range 19–59 years), and 88 were males. A total of 94 patients received full corticosteroid courses, and 68 received reduced corticosteroid courses. The overall response rate (ORR) was 98%. Different corticosteroid dosings had no significant effect on ORR, febrile neutropenia episodes, or hospital admissions. After a median follow-up (mFU) of 58 months, the 5yPFS for the entire cohort was 98% vs. 95% for the standard course vs. the short corticosteroids course, respectively (*p* = 0.37), while the 5yOS was 98% vs. 99% for the standard course vs. short corticosteroids course, respectively (*p* = 0.87). In AS patients intended to be treated with six eBEACOPP cycles, 5yPFS and 5yOS were 100% vs. 97% and 100% vs. 99% for standard vs. short corticosteroid courses, respectively (*p* = 0.56 and *p* = 0.17). In EU patients, 5yPFS was 97% (standard) vs. 95% (short) (*p* = 0.98) and 5yOS 100% vs. 93.3% (*p* = 0.87). Osteoarticular events were numerically lower in patients receiving the shorter prednisone course, both in the whole cohort and in the subgroup of patients treated with six cycles of eBEACOPP, but this difference failed to reach statistical significance. *Conclusions*: eBEACOPP provides excellent and durable first-line disease control. Shortening the corticosteroid course does not compromise efficacy, potentially reducing toxicity. However, longer follow-ups and larger studies are needed for confirmation.

## 1. Introduction

In the past few decades, significant progress has been made in managing classical Hodgkin lymphoma (HL), making it highly curable even in advanced stages.

The two backbone chemotherapy regimens employed in the front-line treatment of Hodgkin lymphoma are ABVD (adriamycin, bleomycin, vinblastine, dacarbazine) and escalated BEACOPP (eBEACOPP; bleomycin, etoposide, adriamycin, cyclophosphamide, vincristine, procarbazine, prednisone). Patients with early stage HL mostly benefit from a combined modality treatment consisting of ABVD followed by involved field radiotherapy [1,2,3]. In advanced-stage disease, studies conducted by groups like the German Hodgkin Study Group (GHSG), Gruppo Italiano per lo Studio dei Linfomi (GISL), and the European Organisation for Research and Treatment of Cancer (EORTC) demonstrated that eBEACOPP is the most potent regimen, providing superior failure-free survival (FFS) compared to baseline BEACOPP (bBEACOPP) or ABVD [4,5,6,7]. While randomized trials failed to prove an improvement in overall survival (OS) [8,9,10,11], a large meta-analysis [12] showed a meaningful OS benefit over less-intensive regimens, with a hazard ratio of 0.74. Furthermore, the GHSG has demonstrated the superiority of two cycles of eBEACOPP followed by two cycles of ABVD (with or without radiotherapy) to four cycles of ABVD with radiotherapy in patients with early unfavorable HL, thus expanding the target population for this regimen to this group [4,13]. However, this benefit is associated with higher rates of severe hematologic toxicity, leading to frequent hospitalization for neutropenic fever and serious infections. Additionally, long-term survivors of HL face an increased risk of lethal secondary cancers and infertility, especially if receiving more than four cycles of eBEACOPP. Notable long-term pulmonary and cardiac toxicities, primarily caused by bleomycin and doxorubicin as well as radiation, are a significant burden to HL survivors, more often seen with higher cumulative doses, such as in patients treated with ABVD [14,15,16,17,18,19,20].

Corticosteroids are used as an integral part of both ABVD and eBEACOPP, either to ameliorate direct regimen-related toxicity, particularly that of bleomycin [21], or as a crucial part of the multi-drug regimen. They can cause a range of side effects, including metabolic disturbances, immunosuppression, and cardiovascular effects. Corticosteroids are well known to impact bone formation and remodeling. Prednisolone administered in doses below 10 mg/day for extended periods can cause osteopenia or osteoporosis and increase the risk of fractures [22,23] in HL patients [24]. Furthermore, steroids are one of the most common factors contributing to the development of avascular hip necrosis (AVN) [25], a debilitating long-term side effect not uncommon in HL survivors [26]. AVN following treatment with eBEACOPP was first reported by Markova et al. [27] from the Czech Republic, followed by similar reports by Israeli [28] and Nordic groups of authors [29]. The GHSG subsequently conducted a comprehensive evaluation of symptomatic AVN on a large cohort of patients [30], thus contributing to a confusingly wide range of reported AVN incidences from less than 1 to 21%.

At our center, we have been using eBEACOPP as a standard front-line treatment for advanced-stage HL since 2009 and for early unfavorable disease since 2014. Following initial observations of symptomatic AVN among our patients, we undertook a study to investigate its incidence as well as causality and found that the use of methylprednisolone, rather than prednisone, was linked to a relatively high incidence of AVN (35.3% of examined patients) [31]. This resulted in a change of practice, with stricter adherence to the original eBEACOPP protocol and exclusive usage of prednisone. Furthermore, since 2016, we started reducing the duration of corticosteroid treatment to 7–10 days instead of the original 14 [32].

The primary aim of this study was to investigate whether eBEACOPP modification with a shorter corticosteroid course impacted efficacy in terms of three measures: the overall response rate (ORR), progression-free survival (PFS), and OS. Second, we aimed to assess whether this intervention reduced side effects, primarily infectious complications and osteoarticular events.

## 2. Materials and Methods

### 2.1. Study Design and Patient Population

We conducted a retrospective study on all patients with newly diagnosed early unfavorable or advanced-stage classical HL who received at least two cycles of eBEACOPP as up-front treatment at University Hospital Centre Zagreb, Croatia, from 2009 until end of 2021. Demographic data, baseline disease characteristics, and detailed information on treatment, corticosteroid type and dosage, outcomes, and adverse events were obtained from patient medical records. Staging was performed according to GHSG criteria [33].

### 2.2. Treatment Regimens

All patients received at least 2 cycles of eBEACOPP, followed by 2 cycles of ABVD for early unfavorable disease or 2–6 cycles of eBEACOPP, bBEACOPP, or A(B)VD for advanced-stage disease. Chemotherapy (eBEACOPP, bBEACOPP, and ABVD) was administered in standard fashion, except for the dosing of corticosteroids, which is the focus of this investigation. Patients in the standard corticosteroid course group were planned to receive 14 days of 40 mg/m^2^ prednisone (or equivalent) per cycle and, in the shorter corticosteroid course, the same daily dose for 7–10 days per cycle. The eBEACOPP regimen was administered with regular G-CSF support, blood counts performed at least twice weekly, and dose reductions according to hematological toxicities. Patients additionally received radiotherapy with 30–36 Gy at the end of systemic treatment to areas in partial remission (PR), with initially bulky disease (>10 cm in maximal diameter) and certain involved extranodal regions (e.g., bone and soft tissues).

### 2.3. Outcome Measures and Statistical Analyses

Efficacy measures included the overall response rate (ORR), PFS, and OS. Response assessment was performed according to Lugano Lymphoma Response Criteria [34]. Toxicity was evaluated retrospectively, according to CTCAE criteria (ver 5.0 2017 [35]).

Patients were divided into subgroups depending on the disease stage (advanced (AS) vs. early unfavorable (EU)), number of planned BEACOPP cycles (2 vs. 4 vs. 6 vs. 8), and duration and type of steroids. Efficacy outcomes were analyzed for the whole cohort and all subgroups. Given the results of our prior work [31], we excluded patients who received methylprednisolone instead of prednisone from the analysis of osteoarticular events. Osteoarticular events of interest were defined as those corresponding to CTCAE [35] grade 3 or higher or those considered clinically meaningful according to intensity of patient symptoms and limitations of everyday living, without other known cause. To avoid bias of different numbers of cycles, we performed a sub-analysis of two uniformly treated cohorts of EU and AS patients. We also evaluated whether the site of the osteoarticular event was within or adjacent to the radiation field to evaluate possible bias of radiotherapy effects.

Survival curves were generated according to the Kaplan–Meier method, while univariate analysis was carried out using the log-rank test with the aid of Excel-based computer program [36]. Correlations between categorical variables were examined using Fisher’s exact test. A *p*-value less than 0.05 was considered statistically significant.

## 3. Results

### 3.1. Patient Demographics and Treatment

After a thorough review of our patient database, we identified 162 patients eligible for this analysis. A total of 33 patients had EU disease at diagnosis, and 129 had AS disease at diagnosis. There was a slight male predominance (88 patients, 55%). The median age at diagnosis was 31 y, ranging from 19 to 59 y. Sixty-two patients (38%) presented with bulky disease. Detailed patient demographics and disease characteristics at diagnosis are summarized in Table 1.

Table 1 shows the demographic details of our patient cohort. Staging was performed according to GHSG criteria, and bulky disease was defined as ≥10 cm.

Among 33 patients with EU, 31 received the 2 planned cycles of eBEACOPP plus 2 ABVD, albeit 2 without bleomycin in the last 2 cycles. Among the AS patients, those treated prior to the middle of 2011 (14 patients, 10.8%) received 4 cycles of eBEACOPP followed by either 4 cycles of baseline BEACOPP (11 patients) or 4 cycles of ABVD (3 patients due to accumulated toxicities of antecedent treatment), which was the current standard at our center. Later on, most AS patients received 6 cycles of eBEACOPP (98 patients, 76%), 1 patient received 6 cycles of eBEACOPP and 2 additional ABVDs, 12 (9%) received 4 cycles of eBEACOPP (as per HD18 trial results [37]), while others received 2 to 5 cycles of eBEACOPP, followed by 1 to 4 cycles of A(B)VD (Table 2). During the eBEACOPP cycles, 94 patients received a full 14-day course of corticosteroids, while 68 patients received a shortened course consisting of 7 to 10 days (median 8 days) of 40 mg/m^2^ of prednisone (or equivalent) per cycle.

Table 2 shows detailed treatment protocols that our patients received. EU patients were scheduled to receive two cycles of eBEACOPP followed by two cycles of ABVD, although one patient received four cycles of eBEACOPP as per the physicians’ discretion. AS patients were scheduled to receive either 4 cycles of eBEACOPP followed by 4 cycles of bBEACOPP, or 4–6 cycles of eBEACOPP. As seen in the table, some of the patients received modified treatment, namely, had certain agents omitted (i.e., bleomycin) or were de-escalated to A(B)VD due to treatment tolerability issues.

### 3.2. Efficacy

The overall response rate among the entire cohort was 98%, with 158 patients (97%) achieving complete remission (CR) and 1 patient (1%) reaching partial remission (PR). Two patients died after receiving two cycles of eBEACOPP, one in the EU cohort and one in the AS cohort. Of 160 patients who finished treatment, 11 patients, mostly before 2011, underwent response evaluation with a CT scan, and 149 patients had PET-CT scans completed. Deauville scores of 1 to 3 were considered PET-negative. Of note, three patients had lesions assessed as Deauville 4 on the final evaluation. Two of these patients underwent a biopsy of PET-positive lesions, which excluded the presence of lymphoma, and have been without signs of disease progression ever since. Therefore, we considered these patients as achieving CR. A single patient had stable disease upon the first evaluation. There was no difference in response rates according to the duration of corticosteroids in the entire cohort and in subgroups defined by stage (*p* > 0.99).

**Figure 1:**
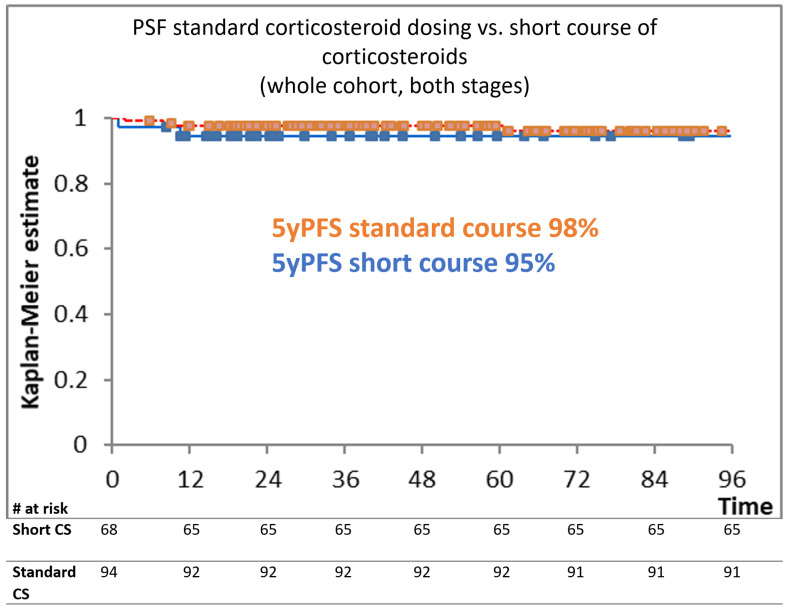
Kaplan–Meier estimation according to different corticosteroid dosing.

After a median follow-up of 58 months, the 5yPFS of patients receiving a standard corticosteroid course was 98% vs. 95% in those receiving the short course (*p* = 0.37) (Figure 1). The 5yOS for the entire cohort was 98% vs. 99% for the standard course vs. the short corticosteroids course, respectively (*p* = 0.87). In the group of 96 patients with AS planned to be treated with six cycles of eBEACOPP after a median follow-up of 62 months, the 5yPFS of subgroups receiving standard versus shortened courses of corticosteroids was 100% versus 97% (*p* = 0.56), respectively. The 5yOS for this cohort was 100% vs. 99% (*p* = 0.17). Similar results were shown for EU patients treated with two cycles of eBEACOPP + two cycles of ABVD; after a median follow-up of 38 months, the 5yPFS was 97% for standard and 95% for a short course of corticosteroids (*p* = 0.98), while the 5yOS was 100% vs. 93.6% for the standard vs. short corticosteroid course, respectively (*p* = 0.81).

Figure 1 shows the Kaplan–Meier estimation curve for PFS in months, depending on the corticosteroid dosing. There was no statistically significant difference between the tested cohorts (*p* = 0.37).

### 3.3. Toxicities

There were no differences between groups with standard and shortened courses of corticosteroids in emergency hospitalization rates (which included febrile neutropenia, CTCAE [35] grade 3 infections, and other acute events) in neither of the tested cohorts (whole cohort, detailed in Table 3). Five patients developed secondary malignancies, two developed AML, one developed breast cancer, one developed prostate cancer, and one developed thyroid cancer. The risk of developing secondary cancer was not correlated to corticosteroid treatment modalities.

Table 3 lists early and late toxicities, depending on the duration of corticosteroid treatment. We did not find any statistical difference between the tested cohorts (Fisher’s exact test). Reasons for hospitalization other than acute infections and febrile conditions included the following: pericarditis, pulmonary embolism, acute cardiac syndrome, and ileus. A total of 25 cases of febrile neutropenia developed after bleomycin administration, including 2 cases of pneumonitis; these patients had bleomycin excluded from later cycles. There were two treatment-related deaths, both in patients who received standard dosing of corticosteroids. Secondary neoplasms were rare and independent of corticosteroid dosing. Two patients developed secondary acute myeloid leukemia (AML), both in the short-course corticosteroid cohort, after initially receiving six cycles of eBEACOPP. Other secondary neoplasms included one patient with thyroid cancer, one with prostate cancer, and one with breast cancer.

The incidence of osteoarticular side effects was evaluated in a cohort of 144 patients, excluding the 18 treated with methylprednisolone instead of prednisone. There was a trend toward a higher incidence of clinically meaningful osteoarticular events in patients receiving the standard course of corticosteroids (6 of 76 patients, 8%) in comparison to those receiving the short course (3 of 68 patients, 4%), but this difference was not statistically significant (*p* = 0.5). To minimize the bias of different cumulative corticosteroid doses depending on the total number of eBEACOPP cycles, we analyzed the effect of different corticosteroid dosings on toxicities among patients who exclusively received six cycles of eBEACOPP and found no statistically significant differences. The incidence of osteoarticular events was also numerically but statistically insignificantly lower in patients receiving six cycles of eBEACOPP with a shorter corticosteroid course (1/35 vs. 4/49, *p* = 0.39). In the entire cohort, we found one patient who developed an osteoarticular side effect relatively near to the radiation field, namely, bilateral humeroscapular periarthritis after receiving irradiation of mediastinum. A detailed description of events by the patient is shown in Table 4. Of note, among patients excluded from this part of the analysis, namely, 18 patients receiving methylprednisolone as part of the regimen, 6 developed AVN.

Table 4 lists osteoarticular adverse events of interest in detail. Only patients receiving exclusively prednisone were evaluated for osteoarticular toxicities (144 patients). There were no statistically significant differences between the tested cohorts. However, there is a slight trend toward a higher incidence of osteoarticular events in patients receiving full doses of corticosteroids.

## 4. Discussion

Given the excellent disease control with contemporary HL treatment, balancing treatment efficacy and toxicity has become a crucial endpoint in most discussions about optimal front-line strategies. Novel agents, such as brentuximab vedotin (BV) and immune checkpoint inhibitors (CPIs), have shown great effectiveness in relapsed/refractory HL [38,39] and are being incorporated into first-line treatments, marking significant progress in the field by reducing exposure to chemotherapy and/or radiotherapy. CPIs are currently only utilized as a part of frontline strategies in clinical trials. The results of the ECHELON trial created the approval of BV in combination with AVD in first-line HL patients, mostly with stage IV disease [40] (the approval was extended to stage III disease by FDA in 2018, and EMA in autumn 2023. However, not all EU countries have followed this extension), meaning these novel agents are still largely unavailable and not reimbursed for most patients in need of a potent front-line therapy, even in the developed countries. This emphasizes the need for further enhancement of treatments we do have at hand.

The eBEACOPP regimen introduced by GHSG has proven to provide powerful disease control, but historical concerns about its acute and late toxicities limited its wider use [41]. In response to these shortcomings, the GHSG sought to reduce eBEACOPP toxicity in the HD12 and HD15 trials by limiting the number of cycles to six and the upper age limit to 60 years, leading to improved results as well as minimized treatment-related mortality and secondary myelodysplasia (MDS)/acute myeloid leukemia (AML) rates [7,42]. Moreover, following the results of the HD18 trial [37], the number of eBEACOPP cycles is further reduced to four in the group of patients achieving PET negativity after two cycles of treatment (PET2). A number of prospective studies tried to avoid administering eBEACOPP to all patients by starting with ABVD and escalating to eBEACOPP only in those who are PET2-positive. However, their results were universally inferior to those achieved with up-front eBEACOPP [43,44,45,46].

At our center, we use eBEACOPP as the front-line outpatient treatment regimen for both EU and AS patients with classical HL younger than 60 years of age. Our results are in concordance with other published data, with 5 yPFS and OS rates exceeding 95% for EU and 98% for AS patients. In our hands, eBEACOPP is a highly effective regimen coupled with significant, albeit manageable, acute toxicities that can be safely used in routine clinical practice. We previously reported on a relatively high incidence of AVN in patients treated with eBEACOPP (35%) receiving methylprednisolone instead of prednisone, despite no differences in cumulative doses of corticosteroids [31]. These findings have prompted a shift in our practice toward strict prednisone use as well as a reduced corticosteroid treatment duration. A similar approach was advocated by the Israeli group that reduced the duration of prednisone treatment to 7 from the original 14 days per cycle of eBEACOPP [47] since 2002, also mainly in order to reduce the risk of AVN. However, it seems that the risk of osteoarticular side effects in HL patients lingers despite the reduction in corticosteroid exposure, as described in their reporting on reduced bone mineral density (BMD) evaluated retrospectively in routine PET-CT scans [24]. They revealed a BMD loss >15% in 48% of patients at disease evaluation after completing therapy. A multi-variate analysis at 6 months post-therapy identified age of ≥30 years and eBEACOPP regimen as significant risk factors for BMD decrease of >15%. Of note, even among patients who received lower cumulative doses of corticosteroids, mostly as antiemetics, the incidence of reduced BMD was vast and calls for reconsideration of corticosteroid usage even as solely supportive therapy. Importantly, none of the patients treated with the eBEACOPP in the Israeli study developed AVN, suggesting that their policy of reducing steroid exposure to one week only is beneficial. This conclusion is consistent with the suggestion by the GHSG to attempt to decrease corticosteroid doses given to HL patients [30].

Our results are similar, even though we could not show a statistically significant difference in terms of a reduction in treatment-related toxicities. Among 68 patients treated with a shorter duration of corticosteroids, 3 developed osteoarticular events grade 3 or higher, of which only 1 was a bone-necrosis event (namely, Morbus Freiberg of right foot). The incidence of similar events was numerically but not statistically significantly higher in the standard duration treatment group, and two patients developed clearcut AVN. One could argue that some of the events not involving bones (e.g., periarthritis humeroscapularis) might not be attributable to corticosteroid treatment at all. However, all these events have developed after the end of HL treatment without other evident causes and were therefore included in our study. An important issue our study did not systematically address was the effect of different corticosteroid dosing on the development of osteopenia and osteoporosis. It would be interesting to know whether a significant number of patients might be spared bone density loss with the administration of shorter courses of corticosteroids. Of note, the GHSG has introduced the new escalated BrECADD regimen, which seems to be as effective as eBEACOPP but with less toxicity [48,49]. The new regimen involves a four-day course of corticosteroids, specifically dexamethasone, which is known for its higher anti-inflammatory activity compared to prednisone. However, the cumulative dose of 40 mg of dexamethasone over four days is equivalent to 1000 mg of prednisone, which is still quite substantial, especially for patients with a lower body surface. It is yet to be seen whether this alteration of the corticosteroid component might influence osteoarticular side effects.

Given the immunosuppressive effects of steroids, we also investigated whether there were differences in emergency hospital admissions due to infections among the standard and short-treatment cohorts and found none. It seems that hematological toxicities caused by myelotoxic substances contribute to infectious complications much more than the duration of corticosteroid exposure.

The ORR in our cohort of patients is high, emphasizing the efficacy of eBEACOPP, and differences in corticosteroid duration did not significantly affect this outcome. Regarding 5-year PFS, this study did not observe significant disparities between the full-dose and short-course corticosteroid groups. This observation extends to the analysis of PFS in specific cohorts, reinforcing the notion that corticosteroid modification does not compromise treatment efficacy in terms of disease control.

Overall, this study contributes to the ongoing discussion surrounding HL management, highlighting the need to minimize treatment-related toxicity without compromising efficacy.

However, it is essential to acknowledge the retrospective nature of the study and its inherent limitations. The size of our cohort and the number of events are rather small, and given it is a real-world analysis, we cannot exclude a possible selection bias and the potential for unmeasured confounders.

## 5. Conclusions

Our study demonstrates that eBEACOPP, administered as a front-line treatment for both EU and AS HL patients, provides excellent disease control. Our results show that reducing prednisone exposure to 8 days instead of the standard 14 days per cycle does not adversely affect the treatment outcome. This was evident in the overall response rate (ORR) among the entire cohort, which was 98%, with no significant differences observed between the standard and short corticosteroid treatment, as well as the 5yPFS, which was comparable between the two groups (98% vs. 95% for standard vs. short course of corticosteroids, respectively). Similar results were observed in sub-analyses of uniformly treated cohorts among EU and AS patients. There was no statistically significant difference in side effects depending on corticosteroid treatment duration, probably due to the size of the cohort and the small number of events. Further studies, preferably in the form of prospective trials and on larger cohorts, are necessary to validate these findings and further refine treatment strategies that balance therapeutic benefit and long-term patient well-being.

## Figures and Tables

**Table 1:** Patient and disease characteristics.

Demographics		No Pts
Total		162
age (y)		
	min	19
	max	59
	median	31
sex		
	male	88
	female	74
stage		
	EU	33
	AS	129
Bulky?	yes	62

Abbreviations: Pts = patients; EU = early unfavorable; AS = advanced stage.

**Table 2:** Treatment characteristics.

Treatment	No of Pts
6eBEACOPP	98
4eBEACOPP	12
2eBEACOPP + 2A(B)VD	31
2eBEACOPP + 4ABVD	1
4eBEACOPP + 2A(B)VD	1
4eBEACOPP + 4A(B)VD	3
5eBEACOPP + 1ABVD	1
4eBEACOPP + 4b(BEA)COPP	11
2eBEACOPP	2
6eBEACOPP+2ABVD	1
3eBEACOPP + 2ABVD + 2AVD	1

Abbreviations: eBEACOPP = escalated BEACOPP (bleomycin, ethoposide, adiamycin, cyclophosphamide, vincristine, prednisone, procarbazine); b(BEA)COPP = baseline BEACOPP (bleomycin, ethoposide, adiamycin, cyclophosphamide, vincristine, prednisone, procarbazine); ABVD = adriamycin, bleomycin, vinblastine, dacarbazine.

**Table 3:** Early and late toxicities (whole cohort, osteoarticular events excluded).

Emergency Hospitalization during Front Line Treatment	Short Course Corticosteroids	Standard Course Corticosteroids	*p* Value
	% (No/Total)	% (No/Total)	
Hospitalization (for any reason)	53 (33/68)	46 (43/94)	0.75
Febrile neutropenia	43 (29/68)	38 (36/94)	0.62
Treatment related death	0	2% (2/94)	/
**Late effects**			
Secondary neoplasms	4 (3/68)	2 (2/94)	0.65

**Table 4:** Osteoarticular adverse events.

Duration of Corticosteroid Treatment	Patient Designation	Description of Events	CTCAE Grade	Comment
**short**	Pt No 19	femoroacetabular impigement, osteitis pubis, sacroileitis, ishiofemoral collision	3	affecting every-day living and limiting self-care
	Pt No 20	Mb Freiberg	3	right foot
	Pt No 68	periarthritis humeroscapularis	2	right sided; had right supraclavicular lymphadenopathy at diagnosis
**standard**	Pt No 78	coxarthrosis	2	billateral
	Pt No 111	AVN	3	hip, right
	Pt No 112	humeroscapular arthrosis	2	billateral, dominantly left, initially had lymphadenopathy in left neck regions
	Pt No 141	tendinitis humeroscapularis; osteoporosis with pathological fractures	3	right hand side, initial lymphadenopathy on both sides of neck
	Pt No 142	AVN	3	hips, billateral
	Pt No 143	periarthritis humeroscapularis	2	billateral, had mediastinal irradiation

Abbreviations: Pt = patient; AVN = avascular hip necrosis; CTCAE = Common Terminology Criteria for Adverse Events.

## Data Availability

The data presented in this study are available on request from the corresponding author.
